# Neuropeptide-S affects cognitive impairment and depression-like behavior on MPTP induced experimental mouse model of Parkinson’s disease

**DOI:** 10.3906/sag-2105-74

**Published:** 2021-07-21

**Authors:** Ayşe ÖZKAN, Mehmet BÜLBÜL, Narin DERİN, Osman SİNEN, Güven AKÇAY, Hande PARLAK, Mutay AYDIN ASLAN, Aysel AĞAR

**Affiliations:** 1 Department of Physiology, Faculty of Medicine, Akdeniz University, Antalya, Turkey; 2 Department of Biophysics, Faculty of Medicine, Akdeniz University, Antalya, Turkey; 3 Department of Medical Biochemistry, Faculty of Medicine, Akdeniz University, Antalya, Turkey

**Keywords:** Parkinson’s disease, neuropeptide-S, cognition, depression-like behavior

## Abstract

**Background/aim:**

The present study proposes to investigate the effect of neuropeptide–S (NPS) on cognitive functions and depression-like behavior of MPTP-induced experimental model of Parkinson’s disease (PD).

**Materials and methods:**

Three-month-old C57BL/6 mice were randomly divided into three groups as; Control, Methyl-4-phenyl-1,2,3,6-tetrahydropyridine (MPTP) and MPTP + NPS 0.1 nmol (received intraperitoneal injection of MPTP and intracerebroventricular injection of NPS, 0.1 nmol for seven days). The radial arm maze and pole tests were carried out, and the levels of tyrosine hydroxylase (TH) were determined using western blotting. A mass spectrometer was used to measure the levels of dopamine, glutamic acid, and glutamine.

**Results:**

The T-turn and time to descend enhanced in MPTP group, while these parameters were decreased by NPS treatment. In the MPTP group, the number of working memory errors (WME) and reference memory errors (RME) increased, whereas NPS administration decreased both parameters. Sucrose preference decreased in the MPTP group while increasing in the NPS group. MPTP injection significantly reduced dopamine, glutamic acid, and glutamine levels. NPS treatment restored the MPTP-induced reduction in glutamine and glutamic acid levels.

**Conclusion:**

NPS may be involved in the future treatment of cognitive impairments and depression-like behaviors in PD.

## 1. Introduction

Parkinson’s disease (PD) is the second most common neurodegenerative disorder after Alzheimer’s disease (AD), and it is distinguished by classic cardinal motor symptoms such as tremor, rigidity, and bradykinesia [ 1].

PD affects about 1% of people over the age of 60 [2]. Nonmotor symptoms of PD include depression, anxiety, emotional and cognitive disabilities [3]. Dementia, working memory, and learning deficits are examples of cognitive dysfunctions [4]. In the early stages of PD, a mean of 26.7% (range, 18.9%–38.2%) of patients have mild cognitive impairment and 20 years after the diagnosis of PD, 80% of these patients have dementia [5,6 ]. Depression, which is considered a risk factor for cognitive dysfunction in Parkinson’s disease, has a clinical significance of approximately 40% in patients with early PD [ 7].

In PD, neurodegeneration is observed in the hippocampus, entorhinal and prefrontal cortex, as well as substantia nigra (SN) [8]. Changes in neurotransmitter systems such as gamma-aminobutyric acid (GABA) and glutamate have been linked to the symptoms (cognitive impairment and depression) occurred in PD [9–11].

The neurotoxin methyl-4-phenyl-1,2,3,6-tetrahydropyridine (MPTP), which selectively damages dopaminergic cells in the substantia nigra pars compacta (SNpc), is widely used to induce PD models in mice and rats [12]. In a previous study, it was observed that MPTP causes impairments in associative memory and elements of affective behavior [13]. MPTP also has an impact on the glutaminergic system and the other neurotransmitter systems [14].

Neuropeptide-S (NPS) is a 20 amino acids peptide neurotransmitter present in the central nervous system (CNS) of vertebrates such as primates, rodents, birds, and amphibians [15–17]. NPS precursor protein has a similar sequence with other sequences including Neuromedin U (NMU) and Neuromedin S (NMS) [18]. The NPS precursor mRNA and Neuropeptide-S receptor (NPSR) mRNA are highly expressed in locus coeruleus (LC), lateral parabrachial nucleus, hypothalamus, thalamus, cortex, and amygdala [15,17 ]. NPSR couples to Gs and Gq proteins and potently increases intracellular calcium levels and cyclic adenosine monophosphate (cAMP) accumulation [ 15,19 ]. As a result, this receptor may have an excitatory effect [20].

NPS has an anxiolytic-like effect and is critical in controlling arousal which is expressed in a neuronal cluster of cells in the LC [15]. Furthermore, NPS administration elevates locomotor activity while decreasing paradoxical (REM) sleep, slow-wave sleep and anxiety-related behaviors [15] as well as food consumption and fear [21–23].

NPS contributes to learning, spatial and contextual memories by mediating glutamatergic neurotransmission enhancement [24]. Zhao et al. found that NPS treatment reversed cognitive deficits in a mouse model of AD by upregulating the levels of postsynaptic density protein 95 (PSD95) and synapsin 1 in hippocampal CA1 neurons [25].

To our knowledge, no research has been conducted into the impact of NPS on cognitive disorders and depression in PD. Therefore, the aim of this study was to examine into the impact of NPS administration on working memory and depression-like behaviors in MPTP induced Parkinsonian mice. The second goal of our study was to investigate and explain the function of glutamate, glutamine, and dopamine in the impairment of working memory in PD.

## 2. Materials and methods

### 2.1. Animals

In this study, three-month-old male C57Bl/6 mice (25–30 g) were used. The animals were purchased from the Akdeniz University Research Unit and were kept in a standard laboratory setting with a temperature of 22 ± 2 °C and a 12-h light-dark cycle. They were given unlimited amounts of food and water. The current study’s experimental protocols were specifically approved by the Institutional Animal Care and Use Committee at Akdeniz University Medical School in Antalya, Turkey (B.30.2.AKD.0.05.07.00/103).

### 2.2. Experimental design

The central NPS injection was applied through intracerebroventricular (icv) cannula implanted chronically. Mice were randomly divided into three groups:

- Control group (received intraperitoneal (i.p.) injection of saline, 0.9% NaCl solution),
- MPTP group (received intraperitoneal (i.p.) injection of MPTP and intracerebroventricular (icv) injection of saline),
- MPTP-injected + NPS treated (received intraperitoneal (i.p.) injection of MPTP and intracerebroventricular (icv) injection of NPS, 0.1 nmol for 7 days, dissolved in 0.9% NaCl solution).

To create the PD model, MPTP was administered 4 times (2 times every day for two days, 4 × 20 mg/kg MPTP) (M0896, Sigma, St. Louis, MO), and the control group received saline with a 12-h interinjection period for two days [26].

**Figure 1:**
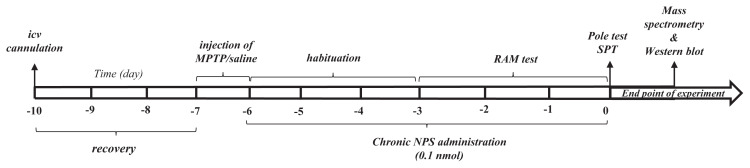
Experimental design. RAM: Radial arm maze, SPT: Sucrose preference test, NPS: Neuropeptide-S.

Mice were habituated to the laboratory and implanted with a cannula in the lateral ventricle. After recovery period, MPTP was administrated for two days and chronic NPS injection (0.1 nmol) was applied for seven days. The radial arm maze test was carried out for four days. At the end of the NPS injection, the pole test and sucrose preference test were performed on day 0. Animals were euthanized and brain samples were collected for biochemical analysis. Figure 1 showed details of the experimental procedure.

### 2.3. Icv cannulation

For the icv injections, the cannula was inserted into the right lateral ventricle (–0.5 mm AP; 1,4 mm ML; 4 mm DV from the bregma). It was fixed by cement and a dummy cannula was placed into the guide cannula to prevent material from entering. To verify that the cannula was located in the correct coordinates, 150 ng human angiotensin-II was administered by icv injection and allowed to access the water. The amount of water consumed by the mice was recorded [27]. Animals that did not consume water within 120 s were eliminated from experimental procotols.

### 2.4. Behavioral test

#### 2.4.1. Pole test

We performed the pole test on the seventh day after the last MPTP injection to assess bradykinesia in the experimental groups. Mice were placed on the top of a pole (diameter 8 mm, height 50 cm, with a rough surface) and allowed to freely explore the pole before falling to the ground (pretrial). After the animals were habituated to the test system, the time it took the mice to completely turn down (T-turn) and descend to the floor (time to descend) was recorded (real trial) [28].

#### 2.4.2. Radial arm maze (RAM)

To measure spatial learning and memory, the radial arm maze (RAM) task was used in mice. The RAM tool consisted of eight arms which have a food region at the end of the arm. The numerous visual objects were fixed on the wall of the maze to orientate itself. Mice were familiarized by exploring the maze for 5 min per day for 3 days. On the first day of habituation, mice were allowed to access food (5 mg chocolate pellet for mice) from all arms before being gradually restrained. Following habituation, each trial was applied twice per day for 4 days. Arms 2, 3, 5, and 7 were consistently baited with one food pellet during each trial, whereas arms 1, 4, 6, and 8 were never baited with food. Each animal was placed in the center of the maze during each trial and testing day, and the working and reference memory tasks were assessed [29]. The maze was thoroughly cleaned and dried before each trial with 70% ethanol.

Three parameters were measured by a video tracking system (Noldus EthoVision XT) in RAM; (i) the number of reference memory errors (RME) (visits to unbaited arms), (ii) the number of working memory errors (WME) (visits to arms already visited in the same trial), and (iii) the accuracy index (number of first entries into the baited arms/total entries into all arms). Reference memory is associated with long-term memory for information that stays consistent through repeated trials (memory for the positions of unbaited arms), while working memory is correlated with short-time memory, in which the information to be recalled changes with each trial (memory for the positions of arms that had already been visited in each trial).

#### 2.4.3. Sucrose preference test (SPT)

Mice were given access to both water and a sucrose solution and their preference for the sucrose solution was quantified [30]. Briefly, the mice were exposed to a 1% sucrose solution for 24 h. After habituation, the water and sucrose bottles were then reintroduced to the mice for 24 h. Before and after the test, the bottles were weighed. The total drinking was calculated as the sum of the water and sucrose bottle consumptions. The sucrose preference was expressed as a percentage of total liquid consumption of sucrose.

After the behavioral tests were completed on the seventh day, the mice were sacrificed, and hippocampal samples were collected for mass spectrometry and SN tissues were taken for western blot analysis.

### 2.5. Protein measurements

A modified Bradford assay with Coomassie Plus reagent was used to determine protein concentration at 595 nm (Pierce Chemical Company) [31].

### 2.6. Western blot analysis

Proteins were extracted from SN tissues with lysis buffer (0.1 M Tris at pH 7.4, 100 × Na-orthovanadate, pH 7.4) supplemented with a protease inhibitor cocktail (P2714; Sigma-Aldrich). The same amount of proteins from each sample were separated on a 10% SDS-PAGE gel, transferred to a nitrocellulose membrane (HATF00010; Millipore) at 4 °C overnight blotting, and hybridized with the primary antibodies tyrosine hydroxylase (TH) (1:1000 dilution; AB113, Abcam, Cambridge, MA, USA) and β-actin (1:1000 dilution; ab16039, Abcam, Cambridge, MA, USA). The membranes were then incubated for 1 h at room temperature with horseradish peroxidase-conjugated secondary antibodies. According to the manufacturer’s instructions, an ECL system (RPN2232; Amersham Biosciences, Buckinghamshire, United Kingdom) was used to detect antibody-bound proteins, which were then analyzed using ImageJ, 1.37v software.

### 2.7. Quantification of dopamine, glutamine and glutamic acid

#### 2.7.1. Sample preparation

The hippocampal tissues were homogenized in a 20-fold volume of a formic acid solution (0.1 M). Homogenates were centrifuged at 18,000 × g for 20 min at 4 °C. The supernatants were collected and kept at − 80 °C until analysis.

#### 2.7.2. Mass spectrometry

The dopamine, glutamine, and glutamic acid standards were provided by Sigma-Aldrich (St. Louis, MO, USA). As previously described, a ultra-fast liquid chromatography (UFLC) combined with mass spectrometry (MS/MS, LCMS-8040, Shimadzu Corporation, Japan) was used [32]. Gradient elution with a flow rate of 0.4 mL/min was used to detect dopamine, glutamine, and glutamic acid. Mobile phase solvent A was water containing 0.1% formic acid and 1% acetonitrile, while solvent B was acetonitrile containing 0.1% formic acid. In positive electrospray ionization (ESI), multiple reaction monitoring (MRM) transitions and responses were automatically optimized for dopamine, glutamine, and glutamic acid. Dopamine, glutamine, and glutamic acid responses were optimized to a linear calibration range of 50 to 1000 ng/mL and a sample analysis time of 4 min [33].

### 2.8. Statistical analysis

The data was presented as the mean ± SEM, and statistical analyses were carried out with the GraphPad Prism software. For the suit with normal distribution, the differences in the pole test, SPT, and mass spectrometry were analyzed using ANOVA followed by Tukey’s posthoc test; the differences in the Western blot were analyzed using Kruskal–Wallis followed by the Mann–Whitney U test. Two-way ANOVA (repeated measure) was used to analyze the RME and WME in RAM, followed by Bonferroni correction. The corresponding p values are shown in the figure legends. The asterisk sign denotes statistical significance between the control and MPTP groups, while the # pound sign indicates statistical significance between the MPTP and MPTP plus NPS groups.

## 3. Results

### 3.1. Pole test

**Figure 2:**
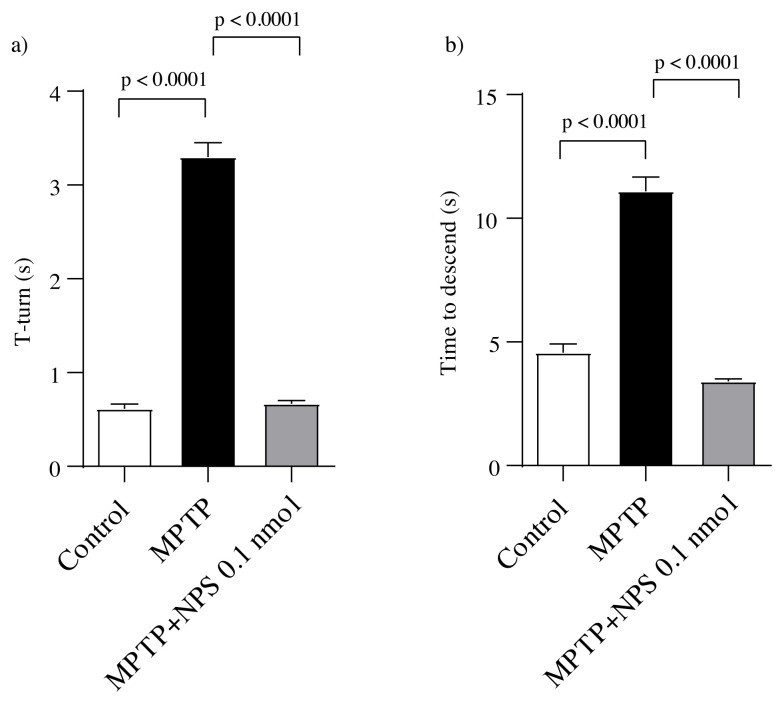
Determination of bradykinesia by pole test. (a) T-turn (s). (b) Time to descend (s). Data are means ± SEM. Statistical analyses are one-way ANOVA followed by Tukey’s multiple comparison test against the indicated group (n = 10).

Motor deficits were expressed using the pole test to investigate the effect of NPS on the behavioral deficits caused by MPTP administration. MPTP administration induced an increase in the descending time and T-turn of mice (p < 0.0001), which was restored by NPS treatment (p < 0.0001). These findings suggest that NPS has neuroprotective properties against MPTP-induced behavioral deficits (Figure 2).

### 3.2. Radial arm maze

**Figure 3:**
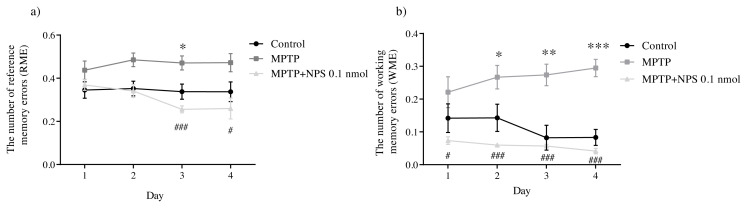
The effect of chronic NPS treatment on memory in radial arm maze task. (a) RME (b) WME. Data are represented as mean ± standard error of the mean. * p < 0.05 vs. Control; ** p < 0.01 vs. Control; *** p < 0.001 vs. Control; # p < 0.05 vs. MPTP group; ###p < 0.001 vs. MPTP group (n = 10).

Figure 3 illustrates reference and working memory errors in different groups. Our observation reveals that the NPS treatment leads in a significant decline in RME when compared to the MPTP group. Altogether, when mice were injected with MPTP, the number of WME increased significantly when compared to controls. As a result of the RAM behavior data analysis, NPS treatment has a positive effect on the MPTP-induced PD model in learning and memory.

### 3.3. Sucrose preference test

**Figure 4:**
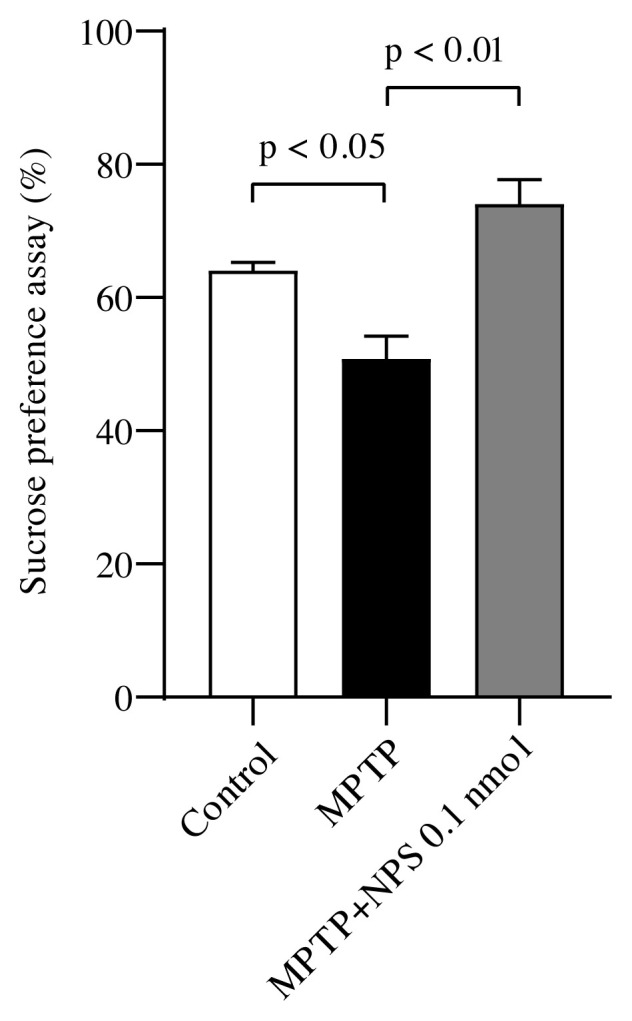
The sucrose preference assay. Values represent means ± SEM (n = 6).

When compared to control animals in the SPT, the MPTP group showed a decreased preference for sucrose (p < 0.05). This effect was significantly reversed by NPS treatment (p < 0.01) (Figure 4).

### 3.4. Western blot

**Figure 5:**
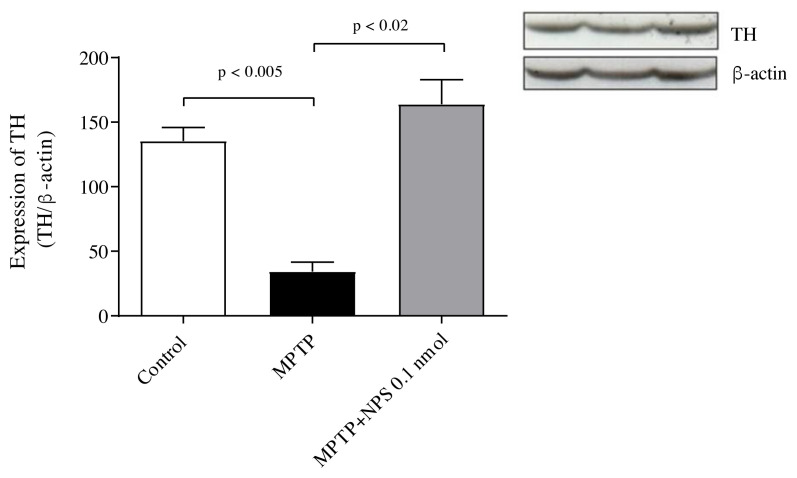
The expression of TH. All data are shown as the means ± standard error mean (n = 6 in each group).

On day 7, there was an increase in the expression of tyrosine hydroxylase (TH) in the SN tissues. MPTP administration caused dopaminergic neuronal death in the SN, but NPS administration suppressed it (p < 0.05) (Figure 5).

### 3.5. Quantitative mass spectrometric measurements dopamine, glutamine and glutamic acid

**Figure 6:**
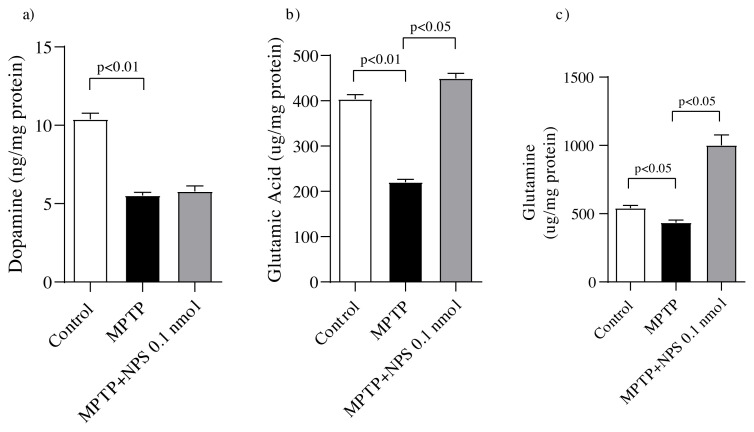
The effect of central NPS treatments on the dopamine, glutamine and glutamic acid concentrations in hippocampal tissues. (a) Dopamine (n = 6), (b) Glutamic acid (n = 6), (c) Glutamine (n = 5). One-way ANOVA followed by Tukey posthoc was used to test the effect of NPS treatments.

After mice were sacrificed and hippocampal samples were obtained, mass spectrometry was used to determine the levels of dopamine, glutamine, and glutamic acid. MPTP caused a remarkable decrease in the levels of dopamine, glutamine and glutamic acid in hippocampal tissues. When compared to the MPTP animals, NPS treatment resulted in a significant increase in glutamine and glutamic acid levels, but not in dopamine level (Figure 6).

## 4. Discussion

As an important endogenous neuropeptide, NPS has been indicated to play an effective role in working memory and depression in a mouse model of MPTP-induced PD. The current study demonstrated that NPS treatment improved the working memory and reduced the depression-like behaviors as measured by RAM and SPT, respectively. Western blot and mass spectrometry techniques were used to support these findings.

Although a variety of neurotoxins, including 6-hydroxydopamine (6-OHDA), paraquat, maneb, and rotenone, are used to mimic the pathological features of PD, MPTP is one of the best models that is most similar to human PD [34]. MPTP is oxidized to MPP+, which alters the permeability of the mitochondrial inner membrane, inhibits complex I of the mitochondrial electron transport chain, and causes ATP depletion in dopaminergic neurons [35]. The C57BL/6 mouse strain is more vulnerable to systemic MPTP than other mouse strains [36]. We preferred to inject MPTP (i.p.) at a dose of 4 × 20 mg/kg every 12 h for 2 days [26]. The primary reason for selecting this dose and method of administration is to reduce the mortality of mice.

Bradykinesia, which is the common symptom and indicator of motor activity in PD, was assessed using a pole test in the current study. According to the findings, MPTP injection increased the descending time and T-turn. However, 0.1 nmol NPS administered centrally has been shown to reduce the severity of bradykinesia. Okamura and colleagues discovered that NPS (icv) treatment reduced inactivity in a dose-dependent manner [ 37]. Furthermore, in our recent study, we have reported that administration of NPS restored the locomotor activity in 6-OHDA induced PD model of rats [33]. These findings explain why central NPS treatment reverses behavioral deficits.

The marker in the identification of dopaminergic neurons is TH, the rate-limiting enzyme in dopamine synthesis, which is known to be diminished in PD and in PD animal models [38, 39 ]. In our study, in the SN, TH expression levels were noticeably reduced in the MPTP group relative to the control group while NPS treatment attenuated the decrease in TH.

In a study conducted by Zhu et al., the levels of DA and 3,4-dihydroxyphenylacetic acid (DOPAC) in the hippocampal tissues were found to be significantly lower in the MPTP-intoxicated PD group [ 40]. In line with Zhu et al.’s findings, in our study, the levels of dopamine in the hippocampal tissue reduced with MPTP injection; whereas, chronic NPS administration caused an increase but did not reach a significant level. MPTP administration increases glutamate efflux in the brain and causes hyperactivity of the glutamatergic system. When glutamate and glycine bind to N-methyl-d-aspartate (NMDA) receptors, they open the channel and cause calcium influx, resulting in neuronal excitation. Therefore, MPTP administration causes neuronal death by increasing glutamate release. In chronic MPTP intoxication, glutamatergic transmission shifts from hyper- to hypoactivity [10]. Although no changes in glutamate and glutamine levels have been observed in PD [5, 11 ], one study found that they differed between PD and control patients [ 9]. In this study, the MPTP administration caused a remarkable decrease in glutamate and glutamine levels. The NPS induced increases in glutamate and glutamine levels were observed but only glutamine levels showed a significant improvement. However, NPS-mediated augmentation of glutamatergic neurotransmission in the amygdala was observed in two previous studies [41, 42 ].

According to our knowledge, SN is the most affected region in PD. Dopaminergic projections are sent to the hippocampus by the SN and the ventral tegmental area (VTA) [ 43]. On the other hand, cognitive disorders such as attention, spatial memory, and learning are observed in PD patients and animal models [44]. The robust impairment of habit learning and spatial working memory were observed in the MPTP model of rats [45,46 ]. MPTP causes dopaminergic neurodegeneration and neuroinflammation in the hippocampus. Neuroinflammation, characterized by microglial activation and cell loss in the hippocampus, leads to cognitive dysfunction associated with dopaminergic degeneration [ 47]. Cognitive deficits in MPTP-treated mice were associated with decreased autophosphorylation of calcium/calmodulin-dependent protein kinase II (CAMKII) in the hippocampus [48]. RAM is commonly used to determine cognitive function in rodents [49]. Our RAM results revealed a significant difference in reference memory errors on the third day between the control and the MPTP group. While in the MPTP group, WME increased significantly on the second, third, and fourth days. Previous studies have shown that intranasal MPTP administration led to significant working memory impairments [50, 51 ]. Thus, these memory deficits observed in PD patients are largely the result of a learning deficit [ 52]. The underlying mechanism of cognitive disorders is the alteration of synaptic plasticity as a result of altered hippocampal LTP. However, LTP is a cellular indicator of synaptic plasticity, learning, and memory. LTP and LTD, two forms of synaptic plasticity, are modulated by endogenous dopamine [53]. Moreover, the decrease of NR2A/NR2B subunit ratio in synaptic N-methyl-D-aspartic acid receptors affects hippocampal LTP [54]. Working memory, which is assessed by RAM, is impaired in PD, and this deficit damages the synaptic integrity of the hippocampus [55]. However, disruptions in other neurotransmitter systems beyond the dopamine underlie some non-motor symptoms of PD [56]. Crabbe and colleagues have reported that the levels of dopamine, serotonin and glutamine were altered in experimental PD [14]. Similarly, our present findings confirm that, when compared to control animals, MPTP significantly decreased glutamate and glutamine levels.

On the third and fourth days, NPS treatment significantly reduced RME. Besides, there was a statistically significant difference in the number of WME between MPTP and the MPTP + NPS 0.1 nmol groups on all days. As a result, both parameters were found to be decreasing with chronic NPS administration. NPS plays an important role in the recall and consolidation of various types of memory, which induces memory enhancement. Retention of recognition memory was significantly prolonged by NPS [23]. NPS also stimulates glutamatergic synaptic neurotransmission [20]. Therefore, all of these findings explain how NPS affects behavioral parameters.

The SPT is used to assess the depression-like behaviors [30]. The depressive-like behavior in animal models of PD, observed in the SPT, was correlated with a reduction in striatal dopamine and hippocampal serotonin content. In this way, the dopaminergic deficit may be linked to this behavior [57, 58 ]. These noradrenergic, serotonergic and dopaminergic changes in the striatal system lead to depression-like behavior in PD [59]. In this study, compared to controls, dopamine level was reduced significantly in mice injected with MPTP. MPTP induced reduction in the sucrose preference ratio was increased in mice received 0.1 nmol of NPS treatment. Therefore, NPS seems to be effective in antidepressant-like behaviors. To regulate behavioral parameters, the NPS system interacts with other neurotransmitter systems. The anatomical distribution of the NPS in the brain determines this interplay [37]. As a result, this study demonstrates that NPS treatment affects cognitive impairments and depression-like behaviors in the experimental mouse model of PD.

## 5. Conclusion

In conclusion, our findings show that NPS has a protective effect in the MPTP-induced Parkinson’s disease mouse model. Impairments of cognitive parameters and behavioral deficits in Parkinsonian mice were recovered by NPS treatment. However, more research is needed to determine the protective mechanism involved in the effect of NPS on cognitive dysfunction and depression in an MPTP-induced mouse model of PD.
